# Supplementary material for: Vasoactive intestinal peptide-expressing interneurons are impaired in a mouse model of Dravet syndrome
Source: eLife. 2019 Jul 8;8:e46846. doi: 10.7554/eLife.46846 (PMC6629374; doi:10.7554/eLife.46846)
Supplement: Transparent reporting form [file elife-46846-transrepform.docx]

***eLife’s* transparent reporting form**

We encourage authors to provide detailed information *within their submission* to facilitate the interpretation and replication of experiments. Authors can upload supporting documentation to indicate the use of appropriate reporting guidelines for health-related research (see [EQUATOR Network](http://www.equator-network.org/%20)), life science research (see the [BioSharing Information Resource](https://biosharing.org/)), or the [ARRIVE guidelines](http://www.plosbiology.org/article/info:doi/10.1371/journal.pbio.1000412) for reporting work involving animal research. Where applicable, authors should refer to any relevant reporting standards documents in this form.

If you have any questions, please consult our Journal Policies and/or contact us: [editorial@elifesciences.org](mailto:editorial@elifesciences.org).

**Sample-size estimation**

- You should state whether an appropriate sample size was computed when the study was being designed
- You should state the statistical method of sample size computation and any required assumptions
- If no explicit power analysis was used, you should describe how you decided what sample (replicate) size (number) to use

Please outline where this information can be found within the submission (e.g., sections or figure legends), or explain why this information doesn’t apply to your submission:

Sample sizes for detecting differences in interneuron excitability were initially estimated to be approximately 20 samples per genotype using a power analysis based on the magnitude of the findings for PV-IN dysfunction in *Scn1a*+/- mice in Favero et al. (2018). Preliminary electrophysiological data analysis was used as feedback to modify total sample collection, including the subdivision of the data into CA and IS groups and by age. For pharmacological experiments using slice physiology, we generally collected between 5-10 cells from at least 3 animals per group in accordance with common practice in the field.

**Replicates**

- You should report how often each experiment was performed
- You should include a definition of biological versus technical replication
- The data obtained should be provided and sufficient information should be provided to indicate the number of independent biological and/or technical replicates
- If you encountered any outliers, you should describe how these were handled
- Criteria for exclusion/inclusion of data should be clearly stated
- High-throughput sequence data should be uploaded before submission, with a private link for reviewers provided (these are available from both GEO and ArrayExpress)

Please outline where this information can be found within the submission (e.g., sections or figure legends), or explain why this information doesn’t apply to your submission:

Electrophysiology data were acquired from multiple slices per mouse, from multiple mice from multiple litters of each genotype. Exclusion criteria are described in the Methods section. For immunohistochemistry experiments, technical vs. biological replicates are explicitly stated in figure legends.

**Statistical reporting**

- Statistical analysis methods should be described and justified
- Raw data should be presented in figures whenever informative to do so (typically when N per group is less than 10)
- For each experiment, you should identify the statistical tests used, exact values of N, definitions of center, methods of multiple test correction, and dispersion and precision measures (e.g., mean, median, SD, SEM, confidence intervals; and, for the major substantive results, a measure of effect size (e.g., Pearson's r, Cohen's d)
- Report exact p-values wherever possible alongside the summary statistics and 95% confidence intervals. These should be reported for all key questions and not only when the p-value is less than 0.05.

Please outline where this information can be found within the submission (e.g., sections or figure legends), or explain why this information doesn’t apply to your submission:

We included raw data of each recorded cell in the majority of figures. Statistical tests are reported in figure legends, and exact *p* values are reported in all cases where direct comparisons are being made. All data are reported as mean +/- SEM as indicated. We report the values of N in each figure legend. Per the Reviewer’s suggestion, all data in Figure 3-Figure supplement 1 is re-analyzed to treat each animal as a N as stated in the manuscript.

(For large datasets, or papers with a very large number of statistical tests, you may upload a single table file with tests, Ns, etc., with reference to sections in the manuscript.)

**Group allocation**

- Indicate how samples were allocated into experimental groups (in the case of clinical studies, please specify allocation to treatment method); if randomization was used, please also state if restricted randomization was applied
- Indicate if masking was used during group allocation, data collection and/or data analysis

Please outline where this information can be found within the submission (e.g., sections or figure legends), or explain why this information doesn’t apply to your submission:

Groups were allocated based on age, genotype, and brain region as described in each figure. Male and female mice were used in equal proportions for all experiments. All analysis was performed with automated matlab scripts blind to genotype.

**Additional data files (“source data”)**

- We encourage you to upload relevant additional data files, such as numerical data that are represented as a graph in a figure, or as a summary table
- Where provided, these should be in the most useful format, and they can be uploaded as “Source data” files linked to a main figure or table
- Include model definition files including the full list of parameters used
- Include code used for data analysis (e.g., R, MatLab)
- Avoid stating that data files are “available upon request”

Please indicate the figures or tables for which source data files have been provided:

The data reported in Figure 3, and the table in Figure 3-Figure supplement 1 are included as an excel spreadsheet. Data from all 258 cells are included for these measurements. This represents the largest data set and core findings of the current study. Additionally, we included annotated matlab scripts of the model which can be used to reproduce Figure 8.
